# Use of an Innovative, Affordable, and Open-Source Short Message Service–Based Tool to Monitor Malaria in Remote Areas of Uganda

**DOI:** 10.4269/ajtmh.2011.10-0528

**Published:** 2011-07-01

**Authors:** Caroline Asiimwe, David Gelvin, Evan Lee, Yanis Ben Amor, Ebony Quinto, Charles Katureebe, Lakshmi Sundaram, David Bell, Matt Berg

**Affiliations:** Foundation for Innovative New Diagnostics, Kampala, Uganda; Foundation for Innovative New Diagnostics, Geneva, Switzerland; Malaria Control Programme, Ministry of Health Kampala, Uganda; World Health Organization, Kampala, Uganda; Global Malaria Programme, World Health Organization, Geneva, Switzerland

## Abstract

Quality health management requires timely and accurate data, and paper-based reporting does not fill this role adequately. The introduction of malaria rapid diagnostic tests and the availability of wireless communications present an opportunity to open direct data transmission and feedback between peripheral health workers and central managers. In November 2009, the Uganda Ministry of Health deployed a short message service–based reporting system in two districts. At a set-up cost of $100/health facility, local technician support of $ 400 per month, and a cost of $0.53/week/clinic, the SMS reporting system was started at more than 140 clinics. Positivity rates for rapid diagnostic tests and artemisinin combination therapy stock outs were 48% and 54% in Kabale and 71% and 54% in Gulu, among other reports, at more than 85% health facilities reporting weekly and without monetary incentives or additional supervision. The SMS-based reporting systems have potential to improve timeliness in reporting of specific, time-sensitive metrics at modest cost, while by-passing current bottlenecks in the flow of data. With the development of specific capacity to manage stock data at district level, the availability of timely data offers potential to address commodity distribution problems and reduce stock-outs.

## Introduction

Malaria management in Africa has long been hampered by poor or untimely and incomplete data on disease incidence and on resource allocation and use. As a result, the planning and implementation of many interventions is hindered and assessment of their impact is poor.^1^,^2^ Despite the advent of major funding initiatives (including the Global Fund to fight AIDS, Tuberculosis and Malaria [GF], the US Presidents' Malaria Initiative, and the projects of the Bill and Melinda Gates Foundation, the availability of data to manage programs effectively, whether on disease incidence, stock levels, or number of patients treated, has remained limited and often of poor quality.^3^,^4^ Data collection usually depends on paper-based systems, which are often laborious, sent late because of transfer costs involved, may be incomplete, and require transcription for electronic data entry and analysis once at district or national level.^5^,^6^

One of the consequences associated with the inherent delays in paper-based reporting reports is frequent stock-outs of health commodities, which can deprive target populations of access to health commodities, potentially leading to an erosion of consumer confidence, demoralized health staff, and damaged credibility of health programs.^7^,^8^ Lack of timely healthcare-related data and lack of transparency in resource management makes it challenging to engage and sustain the support of funding agencies who require performance-based funds management.

Funding to scale up access to accurate, parasite-based diagnosis with rapid diagnostic tests (RDTs) has now made confirmation of malaria diagnosis feasible even at the peripheral levels of the health system, but RDTs have added to the burden of commodity delivery and data collection to support health service delivery in remote areas. Current recommendations by the World Health Organization (WHO) on the universal use of microscopy or RDTs to confirm malaria diagnosis before treatment add urgency to the need to improve data collection and management from the community level of the health system, especially in light of increasing data demonstrating that a significant proportion of malaria-like fevers are caused by other causes.^9^–^13^

In recent years Africa has seen a revolution in communications with the roll-out of affordable wireless services to much of the rural population.^6^ The potential of approaches based on cell phones and mobile devices to address the gaps in field data collection is now widely recognized, and a number of so-called mHealth initiatives have been piloted in low-income settings. Many of these initiatives were documented in the 2009 United Nations and Vodafone Foundation Report on mHealth for Development, which discussed the opportunity of mobile technology for healthcare in the developing world. Although several pilot programs have been published,^14^–^19^ none have yet achieved large-scale use in rural Africa. Doing so will require systems that have minimal or no requirements for new infrastructure; are inexpensive; can be locally maintained; and that make it easy for front-line healthcare workers to make the switch from traditional paper-based reporting systems. It is necessary that new mHealth systems, where possible, fit into existing workflows and be easily modifiable to fit local conditions and to other diseases.

One approach to harnessing the power and reach of mobile phones to support development programs uses reporting systems based on short message service (SMS) or mobile phone text messaging. This approach does not require the installation or maintenance of any software application on the cell phone itself and therefore can be used on even the most basic of cell phones by users in remote areas. Such systems have demonstrated their usefulness in a range of settings, including sharing results of water quality testing initiatives in South Africa; managing the distribution of long-lasting bed nets in Nigeria; supporting education and community empowerment activities in Senegal; and monitoring the nutritional status of children in Malawi, details of which are available (http://mobileactive.org/tagging/rapidsms).

This report describes the introduction into Uganda of an SMS-based malaria reporting system supported by RapidSMS™. RapidSMS™ (www.rapidsms.org), which is based on open-source software (Python™ 3.1.1-2009) (http://www.python.org/) was developed by the United National Children's Fund (UNICEF) in collaboration with the Earth Institute for multiple diseases reporting and commodity tracking.

The project, whose implementation process and output are presented below, is an ongoing collaboration between the Uganda Ministry of Health (MOH), the Foundation for Innovative New Diagnostics (FIND), the Earth Institute at Columbia University, and WHO. The aim was to demonstrate the feasibility (including cost, timeliness, and compliance) of an SMS-based approach to improve data reporting and feedback of key indicators, thus supporting the management of the roll out of RDTs by Uganda's National Malaria Control Program.

## Materials and Methods

### General study area characteristics.

**Figure 1.**
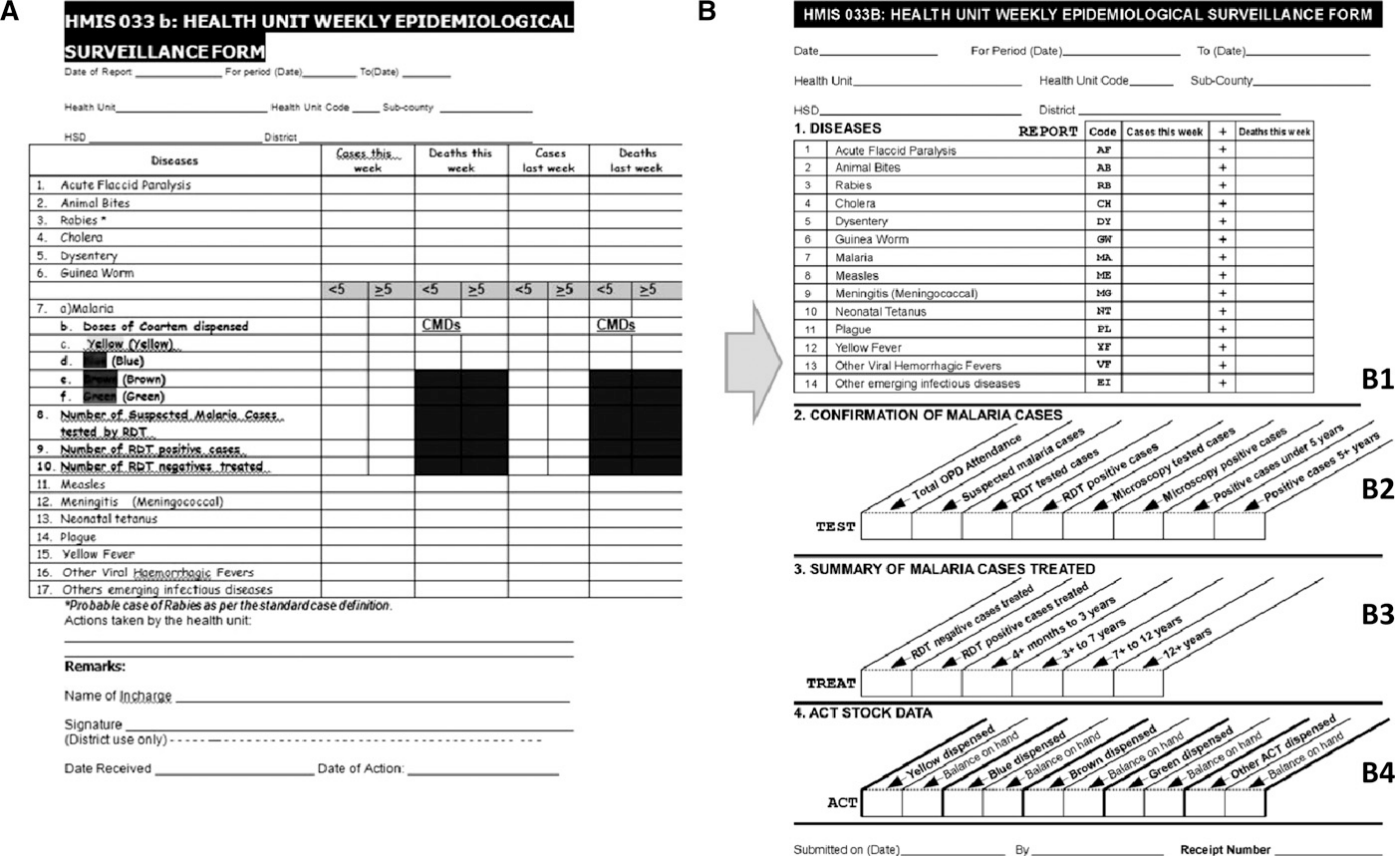
Existing and revised weekly reporting forms, Uganda.

The project was implemented in the Gulu and Kabale districts of Uganda; a country estimated to have a mobile phone coverage of 74% and at least 1 per household (Uganda Bureau of Statistics, unpublished data). The two districts, both distant from the capital of Kampala, are characterized by contrasting malaria prevalences of 63% and 11%, respectively (Uganda Malaria Indicator Survey, 2009). In these two districts, malaria RDTs were introduced by the National Malaria Control Program in 2009 for use by peripheral health centers (Level III serving a parish of approximately 10,000 persons and Level II serving a sub-parish of approximately 5,000 persons). Gulu district includes 43 such health centers, and Kabale includes 104. All health centers in Uganda are required to send weekly reports (HMIS Form 033B), to the district health team headquarters on a number of important indicators covering 14 reportable diseases and including several malaria-specific indicators (Figure 1A).^5^ The weekly reports also require health centers to report artemisinin combination therapy (ACT) stock levels, included in the reporting system because of their relative high expense.

### Designing the SMS-based malaria monitoring platform.

The RapidSMS™ platform is based on the Django web framework and written in Python programming language. It enables rapid development of SMS-driven applications with web-based interfaces for easy monitoring and data analysis. During October–November 2009, the platform was adapted to suit the malaria management program and was customized to suit local requirements, as determined in collaboration with the MOH. This required six person-weeks of programmer time, supported by experts from the Earth Institute and FIND. The only equipment procured for the project was an SMS modem for receiving and transmitting SMS messages and a laptop computer to act as server for the system. A local telecom value-added service provider, DMark Mobile, provided a toll-free SMS phone number, SMS aggregation, and hosted the server. No new financial or other incentives (such as free telephones or air time) were provided to health care workers or other system users. All 336 trained frontline healthcare workers in the two target districts already possessed personal mobile phones, thereby avoiding the expense of purchasing and maintaining mobile devices for the end-users.

### SMS form design.

**Figure 2.**
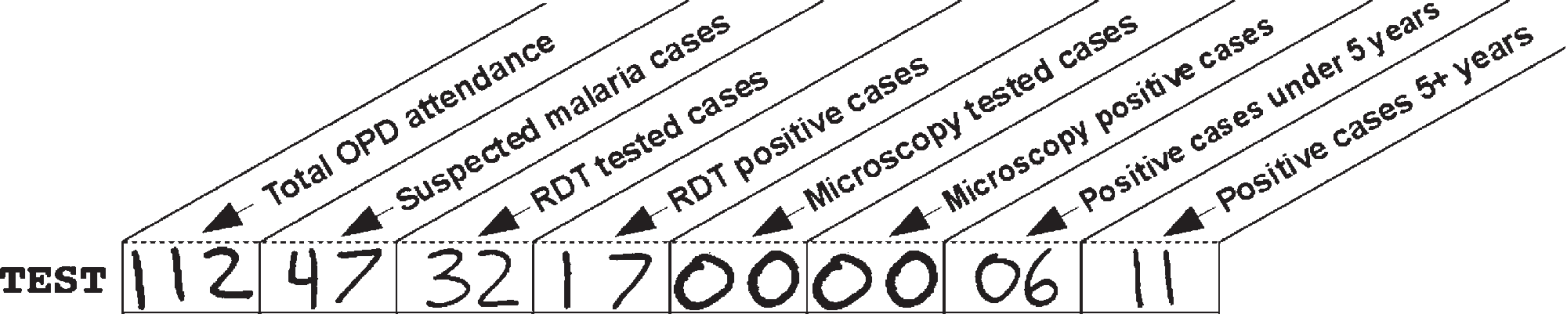
Short message service–based workflows, Uganda.

The initial conception of the project focused on malaria monitoring only, but during consultations between MOH, WHO, and other partners it was agreed to include all current HMIS weekly reporting requirements. The existing weekly reporting form (Figure 1A) was therefore revised to enable healthcare workers to enter health center data in a format compatible with an SMS (Figure 1B). To limit the length of the SMS message while accommodating reporting requirements and to minimize the risk of error, the data were divided into four strings (Figure 1 B2, B3, B4) summarized as “Diseases”, “Test”, “Treat”, and “ACT”, each of which became the header of a single, short SMS message (Figure 2). When completed, each section of the form presents the data in a format that is readily transferable into a simple text message composed of a header, numbers, and spaces. The header acts as a tag, and enables the accompanying figures to be automatically entered into the correct section of a database. The completed paper-based versions of the revised form are retained in the health centers to enable cross-checking of data. During the introductory phase, healthcare workers continued to send in routine paper-based HMIS forms to the district authorities.

Field testing of the modified form was carried out in a district near Kampala to ensure that the data would correspond to that available in health center logbooks, and that the form was user-friendly for front-line healthcare workers.

### Workflow.

**Figure 3.**
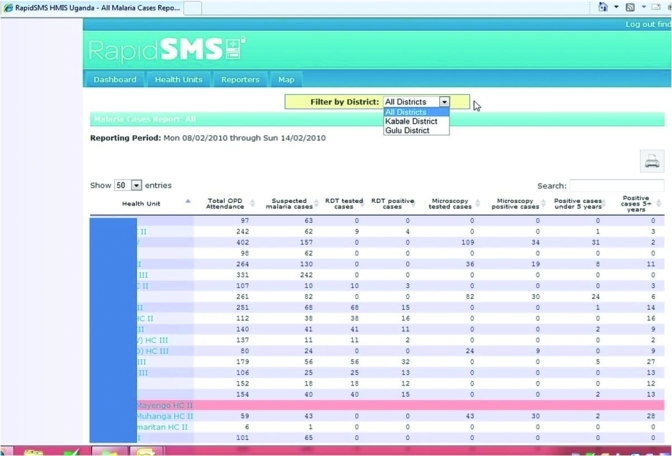
Data from “Test”-related data set, Uganda.

**Figure 4.**
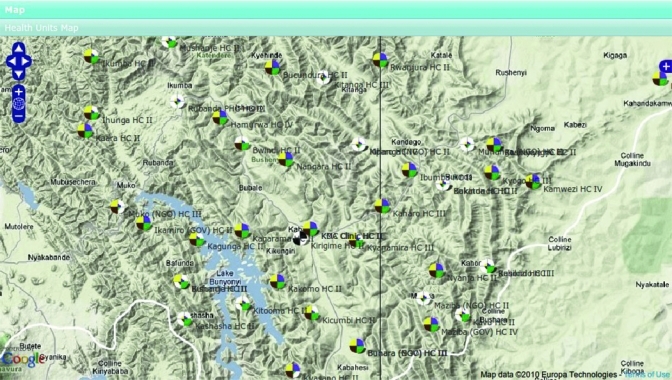
Map of ACT stock availability in four AL color codes, at health centers in Kabale district, Uganda.

**Figure 5.**
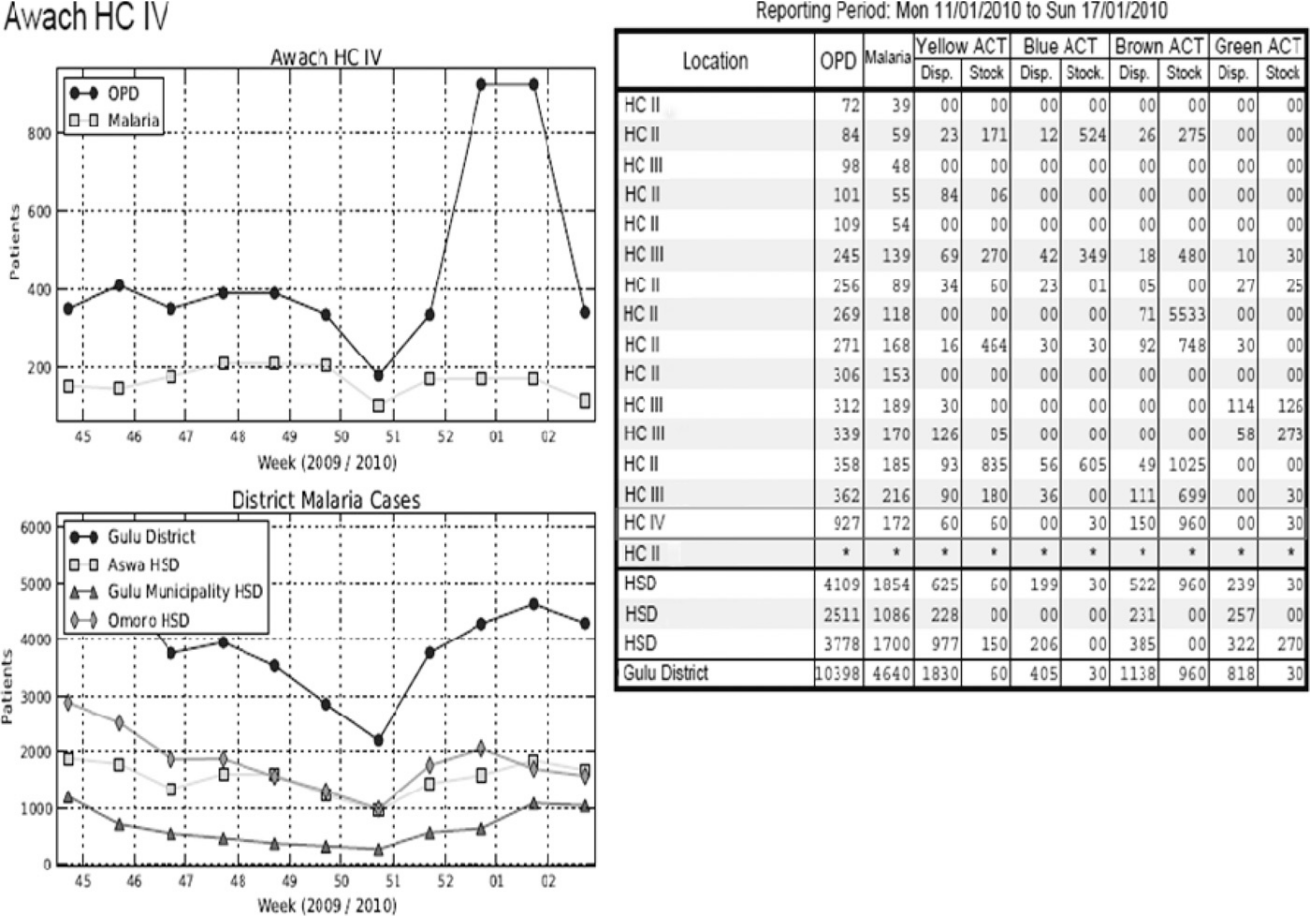
Individual health center report, Uganda.

The work flows and associated feedback from the system incorporated into the use of the RapidSMS™ platform are shown in Table 1. As each message is received, an SMS is immediately sent back via an automated process to confirm that the data has been entered into the system or to indicate an error if data is missing or incorrectly entered. Once all four messages are received, an SMS is automatically sent back to the healthcare worker confirming the information sent, together with a reference number to be transcribed to the clinic paper record to enable later cross-checking of data. If needed, a reminder SMS is sent out by mid-week of the following week if needed to prompt users who have not sent in their data. An optional fifth SMS can also be sent with additional remarks. All SMS are sent to a toll-free number charged directly to the project. Data successfully entered into the system are stored by the central server in Kampala and immediately accessible via a password-protected interface over the internet, viewable in map, table or chart format (Figures 3 and 4). Parameters can be set to control user access so that different user groups only have access to the data that is relevant to their level of responsibility. Contrary to the standard default paper-based HMIS, automated timely reports were available to the district management and surveillance teams for feedback to health centers and district level monthly reporting at no cost (Figure 5).

### Pre-testing the tool.

The SMS-based tool was pretested in four health centers (level II and III), which were located in Wakiso, a neighbouring district to Kampala. Consenting health workers and district health leaders were engaged in the pretest consultative process. The study programmers, coordinator, and ministry of health officials had an interview consultation with the health workers in these health centers that focused on the HMIS 033B form modification, operational procedures for implementation, selected variables and indicators, and suggestions for how to provide incentives to the end user. Pretest outcomes were used to review the training instructions, web interface, and the HMIS 033B form accordingly.

### Planning time and resources.

The study coordinators visited the target districts to engage the district health authorities and laid out plans for implementing the project with quality assurance. The availability of the mobile telephone network signal in the area of study and the availability of at least one handset in each health center was confirmed through a telephone discussion with the health center In-charges. Other discussions on potential implementation risks and their mitigation plans were held at the district health office.

### Training and roll out.

One-day training was conducted for each district. Two frontline healthcare workers, the health center In-Charge and the records focal person, all from each health center, were designated for training on the use of the SMS-based reporting system. Also included were 38 local district opinion and political leaders and health center supervisors to widen understanding of the new system.

At the time of invitation for training, health workers were requested to come with their own mobile phones and an old copy of the clinic's outpatient and laboratory record books in the case of health centers with functional laboratories. There were no exclusion criteria set for the health centers and health workers. Potential risk of introducing the SMS-based system to health workers without mobile phone sets was mitigated by an earlier situation analysis at the district, which sought to establish personal mobile telephone availability at all health centers and willingness to use it for health data transmission.

## Results

### Program set up.

The move from initiation of discussion of concept to actual field implementation took approximately three months. Implementation in Gulu district took place in November 2009 and implementation in Kabale took place in December 2009. The initial investment costs were $50,000 for programmer time and expert technical assistance from the Earth Institute including $600 for the equipment; and $100 in training costs per health facility (predominantly allowances and per diem).

### Network coverage in the pilot areas.

With the exception of only a few clinics, all of the district clinics were within coverage areas and the network coverage was reliable. All SMS messages were tunneled through an SMS aggregator in Kampala that occasionally experienced downtime, but a good average uptime percentage greater than 95% (36 hours of down time per month). In the instances where users were unable to send their reports, they were trained to simply try again the next day.

### Compliance with reporting.

As of August 30, 2010, a total of 5,290 weekly aggregated reports had been sent by 104 of 113 HCs from Kabale and 43 of 45 HCs from Gulu in real time. After training and experience, users could submit all four required messages in less than five minutes. A report consisted of the four required SMS messages alongside routine HMIS-aggregated paper-based reports. This data covered 416,636 outpatient visits and 141,656 suspected malaria cases. Because of a national level stock out of RDTs, which occurred in early 2010, only 48,334 RDT tests were used (positivity rate = 50.2%), and 21,528 microscopy examinations were performed (positivity rate = 39%). Overall compliance with sending the weekly reports (number of completed reports received/number of reports expected) was 88.6% within a one-week cutoff compared with a one month delay considered good practice in paper-based reporting, delivered on the 28th date of the following month.^5^ Although trained, HC IV facilities and hospitals did not participate in SMS-based data transmission because multiple data collection points within each facility would require further work to ensure complete reporting. The cumulative error rate was evenly distributed between clinics (overall failure rate = 8.8%). Errors include messages submitted incorrectly and messages that did not pass built-in data logic tests (e.g., if the number of positive RDTs was greater than the number of RDT tests performed). Errors were mitigated with eight automated responses for each SMS received. Responses were created on the basis of pre-test outcomes.

### Implementation of the open source SMS-based tool.

No health worker requested a telephone, telephone credit, or financial incentives to use the SMS-based reporting system. All participating and non participating HC trainees had mobile phones and agreed to use them to send health data because this process had no financial cost implications. Including the reminder messages, the average weekly SMS messages a registered and reporting health worker receives was 11, in addition to the 4 SMS sent by the same health worker. At 70 Uganda shillings ($.035) per SMS, including charges from the mobile telephone operator and the local value added services provider, weekly mobile data transmission costs for the participating 147 health center IIs and IIIs in both districts were $77/week (or $0.53/health facility/week). These charges also include the hosting and internet connectivity for the server. Additional costs included the hiring of a local technical consultant for system support at $400/month as shown in Table 2.

### ACT stock monitoring and RDT use.

In Kabale district, 94,621 (23.4%) of 404,727 suspected malaria cases were reported by 104 registered HCs level II and III during December 2009–August 2010. Of these cases, only 44.8% were confirmed with the RDT. During the same period, an average 12.1% of the 104 HCs reported total ACT stock out for at least a period of one week. In contrast, a total of 43 HCs levels II and III in Gulu reported 127,074 (47%) of 270,772 as suspected cases of malaria, of which only 4.5% had received RDT-based parasitologic diagnosis. The percentage of reports that indicated a total stock-out of ACT Stock-outs in Gulu were 54.3%. Generally, malaria positivity rate in Gulu was 71% by RDTs (HC II, III) and 48% by microscopy-based blood smear examination in hospitals compared with 48% by RDT and 31% by microscopy in Kabale.

## Discussion

This project shows that it is possible to obtain critical healthcare-related data from remote areas available in real time to help program managers address supply chain issues and to monitor malaria diagnosis and treatment. This was achieved without disrupting normal workflows while also maintaining the current practice of transcribing paper-based reports.

A small initial investment in equipment and programmer time was sufficient to set up the SMS-based reporting system, and running costs were minimal. These findings have the potential for further reduction through engagement with local mobile phone network providers and on-the-job HMIS human resource training. In comparison, the annual expenditure for ACTs in Uganda is projected to be $24 million per year and Uganda's Global Fund commitment for malaria grants (Rounds 2, 4, 7) is $212,100,635 over five years. The system should be highly scalable: additional initial costs for country wide implementation across 100 districts in Uganda would require a further equipment-related expense estimated at $10,000 for an upgraded server, additional SMS modems, and provision for offsite backup; one month of programmer time for further refinement of the coding to strengthen data flow checks and to link the database with the national HMIS system; and additional expenses associated with half a day's training ($100/health center) and $3.0 monthly SMS fees/per health center. Ongoing system support would also require strengthening with an anticipated need to hire a full-time customer services support firm that is familiar with mobile phone technologies and computer systems, but not necessarily a programming expert. The current system benefits from maintaining the paper-based records. If a paper-based system was abandoned entirely, then a backup data transfer system would need to be developed with arrangements for accessing an alternative network.

We found that there was a considerable level of local expertise in working with mobile telephone services, and it was relatively easy to find a local private sector provider who could host the system and assist in the establishment of a toll-free number. Initial acceptance by the MOH and other stakeholders was also facilitated by the flexibility of the platform, which enabled the incorporation of indicators for disease reporting that was not part of the initial plan. The modified HMIS 033B weekly reporting form (Figure 1B) is currently formally adopted by the MOH Resource Center to enable paper-based collection of ACT- and RDT-related data in a linked manner. The familiarity of mobile phone users in Uganda with texting made it straightforward and quick to train the healthcare workers on the system.

The SMS-based reports demonstrate that tracking ACT consumption and RDT use in health centers is now feasible and prompt remedial action is potentially achievable at the district and national level of authority in Uganda. Gaps remain to be addressed in RDT use rates and in addressing stock-outs. The RDT and ACT shortages at national level have contributed to this problem, as well as lack of prompt feedback mechanisms to enable use of data at district and national levels. However, SMS-based reporting of stock elsewhere has demonstrated its capacity to greatly improve ACT stock management and local drug re-distribution. In Uganda, the two pilot districts offer a potential opportunity for better monitoring and impact long term before country-wide scale-up takes place, provided that national stocks are available and district teams are fully engaged.

The report outcomes also suggest that malaria prevalence and ACT stock levels are potential predictors of use of diagnostic services in the public health facilities, as shown by the contrasting reports on the use of parasite-based diagnosis in the context of ACT stock levels in Gulu and Kabale. It is worth noting that the central medical store schedules its supplies of essential medicine to all districts in Uganda on a quarterly basis.

To achieve results in stock management in this pilot study similar to those shown in Tanzania,^17^,^18^ greater efforts would have been initially required to define and document roles and responsibilities, particularly given the new wealth of real-time data available to different individuals within the system. Uganda has a decentralized healthcare system with many responsibilities devolved to the district level. Previously, district health officials had been accustomed to having control over the data sent by health centers before forwarding it to the national level, and there was some apprehension over data being available in the capital, Kampala, at the same time that it becomes available at the district level. This concern prevents the district level managers from adding interpretations of data relevant to its understanding at a central level, but may have advantages in ensuring transparency of stock management and rapidity of reporting. In addition, actions, boundaries, and responses have to be well defined if SMS-based reporting tools are to be adopted by national disease control programs. As one Monitoring and Evaluation Officer in the Ministry of Health expressed it, “Institutionalization is key and the HMIS personnel need to be ones to make sense out of it, if they are to own it. We need to be sensitive to the phobia of “data mining, which is often blamed on partners and hugely affects MOH's acceptability of data-related platforms and strategies.”

Maintaining the observed high compliance may depend on demonstration of a clear improvement in the quality of the support health workers receive as a result of their reports, such as improved stock delivery, in addition to the feedback reports. For example, it was immediately apparent even in this pilot phase that there were large discrepancies in commodity stock levels, and some health centers had excess antimalarial treatments in stocks while others in the same district had none. A clear line of reporting is required to address this, including the malaria focal person, the monitoring and evaluation officer, the district pharmacist, and even national medical stores. In Uganda, the districts health officials did become involved in redistributing artemether-lumefantrine to avoid localized stock outs, but only in an ad hoc manner. Developing a set of MOH guidelines and policies to address these types of issues will be critical to further scale-up of any new data collection system.

Other ongoing challenges include those related to infrastructure: although the use of mobile phones for data reporting via SMS overcomes many of the issues associated with data collection from health centers in remote areas, maintaining internet access and a steady electrical supply is still challenging in remote areas, even at the district headquarters. Addressing these needs and the costs associated with printing of feedback reports for the health centers will be vital to enable effective reporting.

This project benefited from having access to local expertise in programming and in mobile telephone services. This expertise and service is required for a system that uses software that has to be programmed for specific applications. Such expertise may not be as readily available in other resource-limited settings, although the overall level of computer and internet literacy continue to increase throughout Africa, accompanying the expansion of broad-band information communication technology networks continent wide.

Short message service–based messaging platforms offer a valuable opportunity to national disease control programs and Ministries of Health to access specific health data to support evidence-based decision making at several levels of the health system. Our experience with piloting this UNICEF-developed system and findings elsewhere demonstrate that real-time data made available through such a system has the potential to enhance timeliness in reporting health and stock data from remote rural areas. However, timely feedback and stock replenishment should be guaranteed. Ultimately timely transmission of accurate data on malaria incidence should enable better resource allocation and rapid response to changes in transmission. The level of transparency that such an SMS-based system can provide could go far to address the concerns of MOHs and donor partners to ensure that scarce funds are properly used to maximize health impact. Given adequate data management and planning, this inexpensive approach has the potential to greatly enhance national program efficiency and address many of the stock management and disease-monitoring deficiencies of peripheral health services in low-income countries.

## Figures and Tables

**Table 1:** Cost of deploying the short message service–based system for health data monitoring and reporting, Uganda*

Project item description	Units	Cost of unit ($)	Frequency (monthly)	Total cost ($)
SMS text charges from Kabale HCs per week for 11 months	104/113	0.53	4	2,425.28
SMS text charges from Gulu HCs per week for 10 months	43/45	0.53	4	911.6
Local aggregator to manage text volume (DMark)	1	400	10	4,000
Initial technical development (Earth Institute)	4			26,300
Hardware purchase	1	600	–	600
Training (includes trainers, training materials, transport, meals, allowances) for both districts	158	100	–	15,800
Total				50,036.88

* SMS = short message service; HCs = health centers.

**Table 2:** Procedures for sending the short message service–based health data by the health worker, Uganda*

Action carried out by health worker	Report by health worker	Feedback to health worker
Initial registration of the healthcare worker with the system		
Subscription on RapidSMS™ network	“Subscribe- district username”	Success. You are now registered as John Kibuga at DokHC2 with as jkibuga@DokHC3 0772000000.
Weekly reporting		
SMS #1: Send in EPI report, e.g., malaria (ma), dysentery (dy), animal bites (ab), measles (me) by sms string	“Report ma101 dy13 ab4 me1”	Thank you for 02-08/11/09 Diseases Report! MA:101/0 DY:13/0 AB:4/0 ME:1/0
SMS #2: Send TEST report	Test 112 47 32 17 00 00 06 11	2/4 Thank you for 02-08/11/09 Malaria Cases Report! OPD: 112, SUSPECT: 47, RDT: 32, RDT.POS: 0, MICROS: 0, MICROS+: 0, 0-5 POS: 0, 5 + POS: 0
SMS #3: Send TREAT report	Treat 02 17 05 09 10 14	3/4 Thank you for 02-08/11/09 Malaria Treatments Report! RDT.POS: 2, RDT.NEG: 17, 4M-3Y: 5, 3Y-7Y: 9, 7Y-12Y: 10, 12Y+: 14
SMS #4: Send ACT report	Act 14 62 09 00 00 00 21 12 00 00	4/4 Thank you for 02-08/11/09 ACT Stock Data! YELLOW: 14/62, BLUE: 9/0, BROWN: 0/0, GREEN: 21/12, OTHER.ACT: 0/0. Confirmation #20100302
SMS#5 (optional): remarks	E.g., “we have run out of stains for our microscope.”	Action by the supervisor is determined by the need expressed in the Remark.

* SMA = short message service; EPI = epidemiology.
